# Automated analysis of feeding behaviors of females of the mosquito *Aedes aegypti* using a modified flyPAD system

**DOI:** 10.1038/s41598-023-47277-4

**Published:** 2023-11-18

**Authors:** Bianca Monteiro Henriques-Santos, Caixing Xiong, Patricia V. Pietrantonio

**Affiliations:** Department of Entomology, Texas A&M University, College Station, TX 77843-2475 USA

**Keywords:** Behavioural methods, Neurophysiology

## Abstract

Mosquitoes present a global health challenge due to their ability to transmit human and animal pathogens upon biting and blood feeding. The investigation of tastants detected by mosquitoes and their associated feeding behaviors is needed to answer physiological and ecological questions that could lead to novel control methods. A high-throughput system originally developed for research in fruit flies feeding behavior, the flyPAD, was adapted and tested for behaviors associated with the interaction or consumption of liquid diets offered to females of the mosquito *Aedes aegypti* Liverpool strain. Females were given water, sucrose solution and sheep blood in choice and non-choice assays. The volume ingested was evaluated with fluorescein. The placement of the system on a heated surface allowed blood consumption, and without females puncturing a membrane. The flyPAD system recorded nine feeding behavioral variables, of which the number of sips and number of activity bouts correlated with meal volume ingested for both sucrose solution and blood. The adaptation to mosquitoes of the flyPAD system differentiated feeding behavior variables between two feeding deterrents, capsaicin, and caffeine. The flyPAD has potential to quickly assess diverse tastants in both sucrose and blood and may contribute to characterizing more precisely their mode of action.

## Introduction

The yellow fever mosquito, *Aedes aegypti* (L.) is the primary vector of emerging and reemerging arboviral pathogens that cause human diseases including yellow fever, Zika, dengue fever, and chikungunya^1^. The current distribution of *Ae. aegypti* is the widest ever recorded for this species, increasing public health concerns about new disease outbreaks^2,3^. While *Ae. aegypti* mosquitoes of both sexes feed on plant nectar or similar sugary solutions^4^, only the females bite and feed on blood, preferentially human, and it is during biting that an infected female can transmit these pathogens. Due to the lack of vaccines and curative treatments for the aforementioned diseases, vector control with insecticides is still the primary strategy to combat mosquito-borne diseases, causing the concomitant increase in insecticide resistant mosquito populations^5–9^.

Exploiting the chemosensory system (olfactory and taste or gustatory) of *Ae. aegypti* mosquitoes for surveillance^10^ and/or control is an alternative strategy that has gained further momentum especially after the availability of an improved mosquito reference genome, and the discovery of novelties in its olfactory system that are different from those of *D. melanogaster*^11–14^. Multiple chemosensory receptor genes belonging to different families can be expressed in a particular *Ae. aegypti* sensory neuron enabling some of these sensory neurons to respond to multiple olfactory cues^12^. It is foreseen that “push–pull” strategies similar to those applied to agricultural pests^15^ could be useful to control mosquitoes by pushing them away with environmental repellents and attracting them to deadly baits or traps^16^. Dengue and malaria vector control interventions targeting the chemosensory system through attractive toxic sugar baits (ATSB) can potentially reduce transmission by reducing feeding rates^17,18^. Furthermore, baits can also be coupled with anti-parasitic compounds, leading to reduced transmission^19^.

Less is known about the taste system than the olfactory system in *Ae. aegypti,* however, taste is an area of active research for the possibility of using toxic and/or behavioral-changing baits targeting female gustatory receptors (GRs)^20^ or other receptors in legs and mouthparts^21^. Phagostimulants are considered for this purpose^22,23^. In *Ae. aegypti*, gustatory receptors and their relative transcript expression were reported in female leg tarsi and labella^24^. The leg and labella, along with the mosquito antennae, are covered by specialized sensory hairs, the sensilla, which are typically innervated by two or three olfactory sensory neurons^25^. The transcriptome of the maxillary palp of mature *Ae. aegypti* females revealed gustatory receptor genes of yet unknown function, as uniporous taste sensilla are not present in these appendages^26^. This species also tastes the repellent DEET through the legs by contact^27^.

Although in nature *Ae. aegypti* females have become highly anthropophilic, feeding almost daily on human hosts, and rarely feeding on nectar^28,29^, sugar can still be found in detectable amounts in the gut of these mosquitoes^30^. Sugar feeding plays an important role for reproductive maturation, due to its impact in the production of juvenile hormone (JH), a hormone that plays a key role in insect reproduction^31^. JH synthesis occurs at rates that are proportional to the supply of acetyl units acquired with a sugar meal^32^. In this way, females fed a high sugar meal have significantly higher titters of hemolymph JH compared to mosquitoes fed low sugar meals^33^. Understanding the propensity to sugar feed is also fundamental, given that some novel mosquito surveillance and control strategies rely on sugar baits^34,35^. Nectar feeding constitutes the major source of nutrition for males. Consumption of sugar solutions with or without other added chemicals in *Ae. aegypti* is commonly assessed by capillary feeder assays (CAFE)^21,36^. The CAFE assays had been originally developed for adult *Drosophila melanogaster* fruit flies^37^.

In the context of blood feeding, the stylet is the key appendage that is specialized to detect blood and a subset of stylet neurons are tuned to specific blood components, namely ATP, NaCl, glucose and NaHCO_3_^38^. With respect to previous attempts to evaluate blood feeding behavior, a “bitometer” was developed to electronically determine the penetration of the host skin^39,40^. Most recently a study utilized human subjects to define *Ae. aegypti* biting modalities using electropenetrography (EPG)^41^ and a “biteOscope” that tracks mosquito feeding behavior through imaging was reported^42^. These systems do not offer a complete assessment of both probing and actual blood volume consumed as to be useful to screen blood feeding deterrents.

With the goal of assessing feeding deterrents and adding precision to measurements of liquid food consumption in females of *Ae. aegypti* beyond CAFE assays we adapted and tested the flyPAD system^43^ to investigate both sugar and blood feeding modalities. The flyPAD system, developed for feeding choice assays with fruit flies, utilizes changes in capacitance to record the contacts of the mouthparts (or legs) with the diet and produces precise traces for different behaviors during feeding^43^. Herein, feeding recordings obtained with the flyPAD system were coupled with the addition of a fluorescent dye in the liquid diets to validate the flyPAD outputs of several feeding variables when sugar solution or blood were offered to *Ae. aegypti* females. We demonstrated that a minimal structural flyPAD modification to fit the height of adult mosquito females enabled us the measurement of ingested sugar solution and associated feeding behavior variables in choice and non-choice assays. In addition, when the flyPAD system was placed over an external heating device it successfully allowed female blood feeding and the characterization of blood feeding behavioral patterns and volume ingested in non-choice assays. In the context of blood feeding the flyPad system was validated with known feeding deterrents for its future use in screening such feeding deterrents even in the presence of ATP in blood as phagostimulant.

## Results

**Figure 1:**
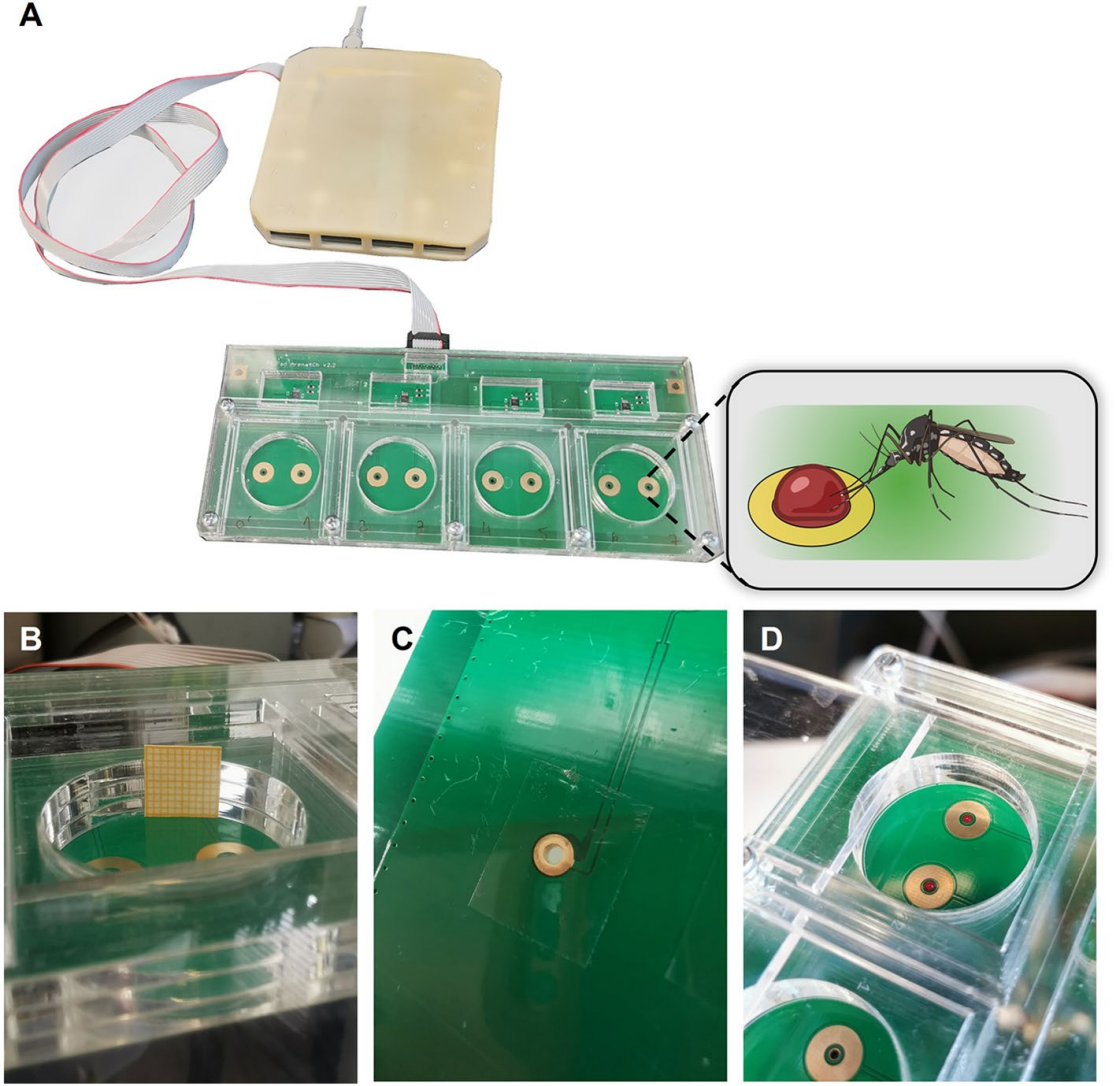
(**A**) The flyPAD setup: the behavioral chamber is composed of four arenas; each containing two channels (golden flat rings containing blood) and is connected to the multiplexing board which collects the signals and sends them to a computer via a USB. The inset shows the graphic of a female feeding blood from one channel. (**B**) Arena of a modified chamber for mosquito assays: the arena is approximately 8 mm in height and is shown here with a 1 cm^2^ of millimeter yellow paper for height reference. Note the three layers of plexiglass used instead of the normal two layers in the original chamber shown in (**A**). (**C**) Close-up of the sealed bottom of a channel using 1 cm^2^ piece of adhesive film for qPCR plates. (**D**) Arena containing two channels, each with 4 µL of blood.

In this study, we successfully adapted the flyPAD system, which was originally designed for adult *Drosophila*, to characterize feeding behaviors in female *Ae. aegypti* mosquitoes on either sucrose solution or blood (Fig. 1A). The chambers were modified in height to accommodate the standing mosquitoes by adding an extra plexiglass plate of approximately 2 mm, making the arenas 8 mm tall (Fig. 1B). Despite the system being developed for agar-containing diets, we adapted it to accommodate liquid meals by closing the bottom of wells with qPCR plates adhesive seal film pieces (Fig. 1C). In this way, we were able to add up to 4 µL of the liquid meal in each well (Fig. 1D), and chambers were placed on top of a heated surface, to keep meals warm.

**Figure 2:**
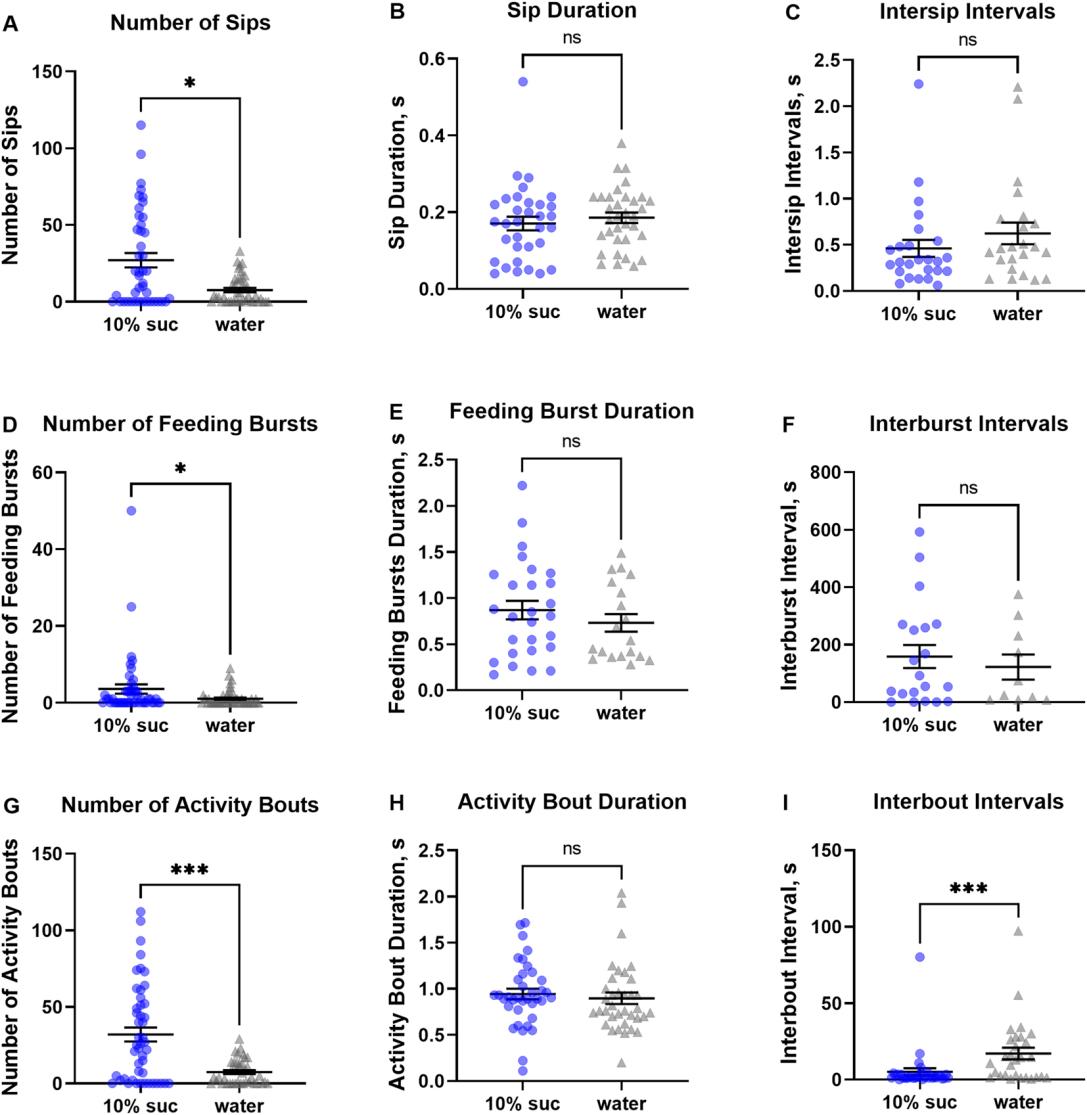
Comparison between feeding behaviors of female *Aedes aegypti* when offered 10% sucrose solution (circles in blue, 10% Suc) or water (triangles in gray) in choice assays using the flyPAD system with one female per arena. (**A**) Number of sips. (**B**) Duration of the sips, in seconds (s). (**C**) Duration of intersip intervals (s). (**D**) Number of feeding bursts, each characterized as three or more consecutive sips. (**E**) Duration of each feeding burst. (**F**) Duration of interburst intervals (s). (**G**) Number of activity bouts, indicating how often the mosquito approaches the food. (**H**) Duration of the activity bouts (s). (**I**) Duration of interbout intervals (s). Symbols represent recordings from individual mosquitoes during 30 min observation period, horizontal lines are means (n = 48) ± standard error of the mean (SEM). Mann–Whitney test, asterisks denote a statistical significance, where one asterisk (*) indicates *P* < 0.05, three asterisks (***) indicate *P* < 0.001, and not significant (ns) indicates *P* > 0.05.

The choice assays between sugar solution and water shown in Fig. 2 were performed to evaluate the feasibility of using liquid meals in the flyPad system because its channels were originally developed to provide meal treatments to fruit flies in 1% agarose. For the evaluation of liquid diets, first the starved females of *Ae. aegypti* were set to choose between a 10% sucrose meal and water provided within the same arena. Our results demonstrated that not only the system is adaptable to the use of liquid meals instead of gel form, but also that the system differentiates outputs of different variables corresponding to mosquito feeding behavior. In addition, these outputs were different for each channel when the females were offered different meals in choice assays. Females starved from any macronutrients for 24 h but given access to water, preferred to feed on a sugar solution over drinking water, as it would be expected. This can be observed in the significantly greater number of sips recorded for females in the channels containing the sucrose solution (S) than water (W) (S = 27.07 ± 4.7 vs. W = 7.59 ± 1.39) (Fig. 2A, P < 0.05). There were no differences in sip duration (S = 0.17 ± 0.02 vs W = 0.18 ± 0.01) (Fig. 2B) or intersip intervals (S = 0.46 ± 0.09 vs W = 0.62 ± 0.11) (Fig. 2C). The same significant preference for 10% sucrose was observed in the number of feeding bursts (S = 3.62 ± 1.2 vs W = 1.08 ± 0.29) (Fig. 2D, P < 0.05), although burst duration (S = 0.87 ± 0.1 vs W = 0.73 ± 0.1) (Fig. 2E) and interburst intervals (S = 158 ± 40 vs W = 122 ± 44) did not differ between diets (Fig. 2F). As females performed more feeding bursts on the sucrose channel, this behavior resulted in a higher number of activity bouts (S = 31.96 ± 4.59 vs W = 7.44 ± 1.24) (Fig. 2G, P < 0.001), however, there were no significative differences for the duration aspect of activity bouts (S = 0.94 ± 0.06 vs W = 0.9 ± 0.06) (Fig. 2H). Therefore, the choice of feeding on sugar or water did not affect how long the mosquito interacted with the meals for sips, feeding bursts, or activity bouts (Figs. 2B,E,H). Correspondingly, the interbout intervals were longer for channels with water only (S = 5.09 ± 2.34 vs W = 17.09 ± 3.82) (Fig. 2I).

**Figure 3:**
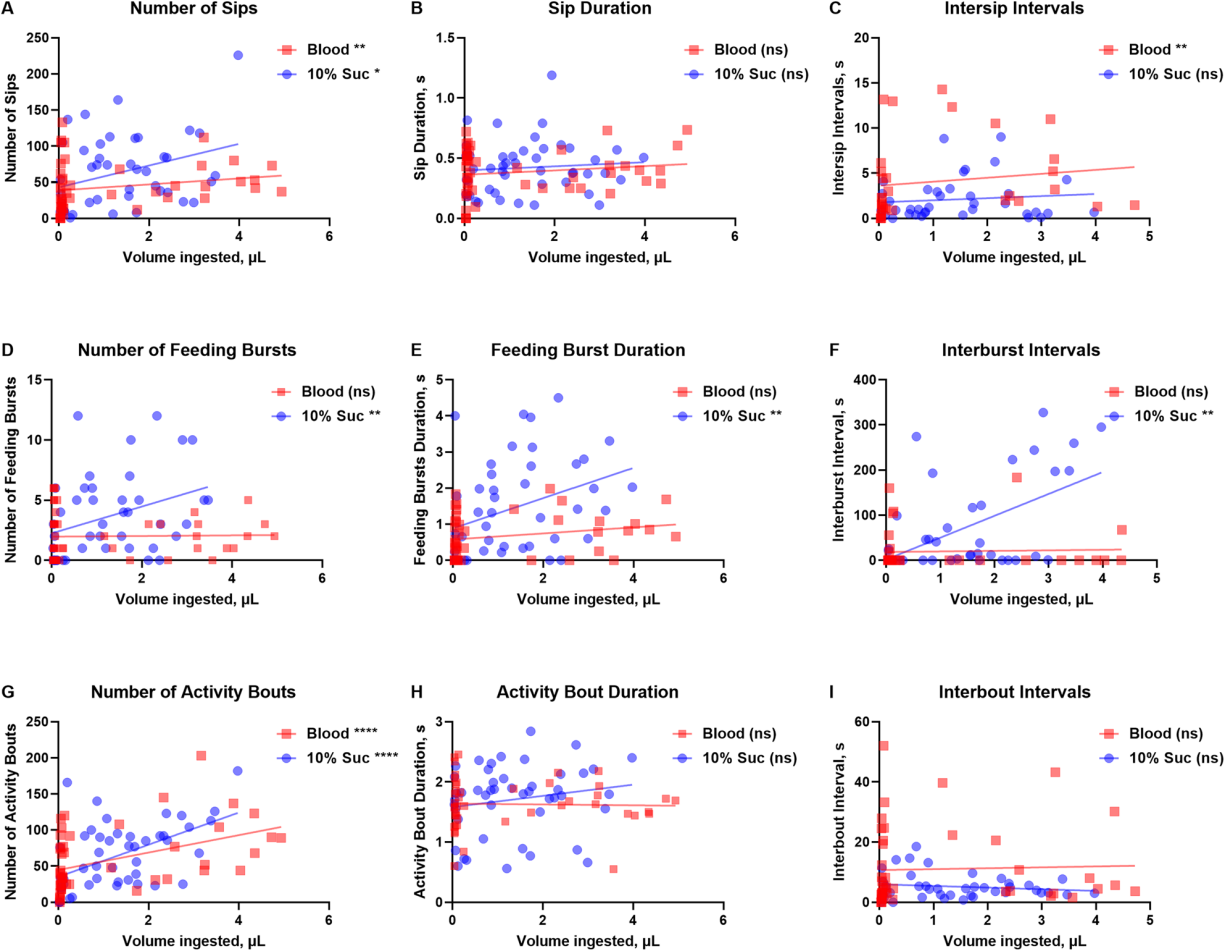
Comparison between blood (squares in red) and sucrose (circles in blue—10% Suc) correlations of data collected by flyPAD within each arena and the volume of each meal ingested by single females of *Aedes aegypti*. Non-choice assays were with single females placed in arenas containing either blood or sucrose in the two channels. The volume ingested was estimated by fluorescence measurements. (**A**–**I)** Correlation analyses between flyPAD output variables and the respective meal volume ingested. (**A**) Number of sips. (**B**) Duration of sips in seconds (s). (**C**) Intersip intervals in seconds. (**D**) Number of feeding bursts. (**E**) Duration of feeding bursts in seconds. (**F**) Duration of interburst intervals. (**G**) Number of activity bouts. (**H**) Duration of each activity bout in seconds. (**I**) Interbout intervals in seconds. N per treatment = 48 females. Nonparametric Spearman correlation test (two-tailed); asterisks denote a statistically significant correlation between data, where one asterisk (*) indicates *P* < 0.05, and four asterisks (****) indicates *P* < 0.0001, and not significant (ns) indicates *P* > 0.05. R stands for the Spearman correlation coefficient (rho). See Table 1 for r values, *P* values and number of observations.

Following these results, we wanted to evaluate whether, and if so, which of the flyPAD outputs for the different feeding modalities previously defined for fruit flies, namely sip, burst, bout, their respective duration and the duration of their intervals as in a previous study^43^, correlated to the actual meal volume ingested by the female mosquito. These correlations were considered crucial to validate the system for research on feeding stimulants or deterrents. For this, we performed non-choice assays in which females were offered in both arena channels either sucrose solution with fluorescein, or blood containing ATP as phagostimulant and fluorescein. Notably, the mosquitoes were able to successfully feed directly on an open pool of blood without the necessity of a membrane. Since meals in both channels would be supplemented with the same fluorophore a non-choice array was necessary to compare the volume ingested between the two diets. Meal volumes were estimated using the fluorescence of the mosquito homogenate and a standard curve. These volume data were plotted against their respective flyPAD output data (Fig. 3), and their correlations along with their significance (*P* values) were calculated using GraphPad Prism software and are shown in Table 1. For both sucrose and blood meals, the number of sips (Fig. 3A) and the number of activity bouts (Fig. 3G) positively correlated with the volume ingested (Table 1, columns to the left). For sip number, the Spearman correlation coefficient (ρ = r) r was 0.339 for sucrose and was 0.396 for blood (Table 1, top panel). The best correlation was observed for the number of activity bouts for both sucrose solution and blood, for which r was 0.534 and 0.546, respectively (Fig. 3G and Table 1, bottom row). Regarding the number of feeding bursts, feeding burst durations, and interburst intervals, these flyPAD outputs only correlated significatively with the volumes ingested by the females offered sucrose (Figs. 3D–F, respectively; Table 1). On the other hand, intersip intervals was the only variable that significatively correlated with blood meal volumes ingested by the mosquitoes (Fig. 3C) but not for volume of sucrose fed. Output variables that did not correlate with the estimated meal volumes for either sucrose of blood meals were sip duration (Fig. 3B), activity bout duration (Fig. 3H), and interbout intervals (Fig. 3I) (Table 1).

**Table 1:** Correlation analysis of flyPad output variables and estimated ingested meal volume of single *Ae. aegypti* females using a fluorescence indicator in the offered 10% sucrose solution of blood containing ATP.

	Correlation: volume ingested (μL) vs. Sip outputs					
	Number of sips		Sip duration		Intersip intervals	
	10% Suc	Blood	10% Suc	Blood	10% Suc	Blood
Spearman r	0.3389	0.3951	0.0813	0.1511	0.1321	0.4564
P (two-tailed)	0.0244	0.007	0.5827	0.3105	0.4293	0.0076
P value summary	*	**	ns	ns	ns	**
Number of XY pairs	44	46	48	47	38	33

	Correlation: volume ingested (μL) vs. Burst outputs					
	Number feeding bursts		Feeding bursts duration		Interburst intervals	
	10% Suc	Blood	10% Suc	Blood	10% Suc	Blood
Spearman r	0.3721	0.1247	0.4205	0.2585	0.4783	0.0139
P (two-tailed)	0.0152	0.4037	0.004	0.0903	0.0014	0.9347
P value summary	**	ns	**	ns	**	ns
Number of XY pairs	42	47	45	44	42	37

	Correlation: volume ingested (μL) vs. Bout outputs					
	Number activity bouts		Activity bouts duration		Interbout intervals	
	10% Suc	Blood	10% Suc	Blood	10% Suc	Blood
Spearman r	0.5336	0.5451	0.1881	0.0475	0.0192	0.2463
P (two-tailed)	< 0.0001	< 0.0001	0.2055	0.7567	0.9053	0.1071
P value summary	****	****	ns	ns	ns	ns
Number of XY pairs	48	47	47	45	41	44

**Figure 4:**
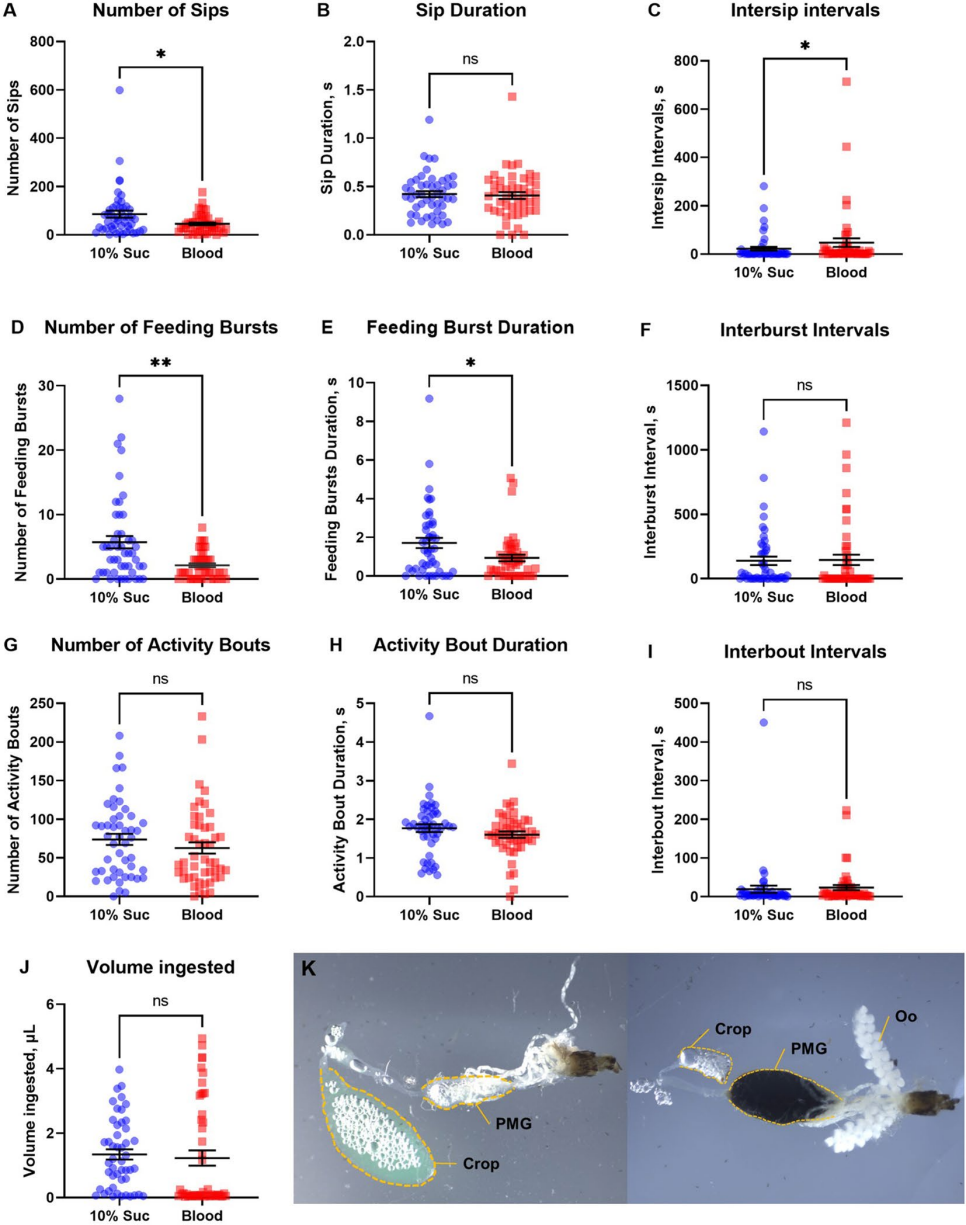
Comparison between feeding behaviors of female *Aedes aegypti* when offered blood (squares in red) or sucrose (circles in blue, 10% Suc) using the flyPAD system in non-choice assays. (**A**) Number of sips. (**B**) Duration of the sips, in seconds (s). (**C**) Duration of intersip intervals (s). (**D**) Number of feeding bursts, each characterized as three or more consecutive sips. (**E**) Duration of each feeding burst (s). (**F**) Duration of interburst intervals (s). (**G**) Number of activity bouts, indicating how often the mosquito approaches the food. (**H**) Duration of the activity bouts (s). (**I**) Duration of interbout intervals (s). (**J**) Total volume ingested by each female. Data are the same as shown in Fig. 3. Symbols represent outputs from individual mosquitoes, lines are means (n per treatment = 48) ± standard error of the mean (SEM). Mann–Whitney test, asterisks denote a statistical significance, where one asterisk (*) indicates *P *< 0.05, and two asterisks (**) indicates *P* < 0.01, and not significant (ns) indicates *P *> 0.05. (**K**) Differential tissue distribution of sucrose solution and blood in the female digestive system. Left panel: the sucrose solution is directed to the crop that is enlarged and colored green by fluorescein. Right panel: the blood meal is only directed to the gut, where it colors the enlarged posterior midgut (PMG) deep brown; female was dissected after 24 h of the blood meal containing fluorescein. Notice the clear and smaller crop than in the left panel, and the grown white vitellogenic oocytes (Oo). Tissues in both panels were delineated with yellow dashed lines to facilitate visualization. Photographs were obtained at 25 × magnification under a stereomicroscope Olympus SZ61 (Tokyo, Japan) mounted with an Infinity 5 camera (Teledyne Lumenera, CAN).

Subsequently, the same flyPAD data outputs shown in the correlations in Fig. 3 were analyzed to compare mosquito feeding behaviors when offered sucrose solution or blood in both channels of the arena (Fig. 4). Female mosquitoes interacted more often with the sucrose solution (S), compared to blood (B). This can be observed in the significantly higher number of sips (S = 85 ± 14 vs B = 45 ± 5.5) (Fig. 4A), of feeding bursts (S = 5.7 ± 0.94 vs B = 2.1 ± 0.3) (Fig. 4D), and also in the longer duration of these bursts (S = 1.7 ± 0.27 vs B = 0.93 ± 0.17) (Fig. 4E). There were no statistically significant differences in the duration of the sips (S = 0.42 ± 0.03 vs B = 0.41 ± 0.04) (Fig. 4B), duration of interburst intervals (S = 1.39 ± 33 vs B = 1.46 ± 41) (Fig. 4F), in the number of activity bouts (S = 74 ± 7.1 vs B = 63 ± 7.3) (Fig. 4G), the duration of the bouts (S = 1.8 ± 0.1 vs B = 1.6 ± 0.1) (Fig. 4H) or of the interbout intervals (S = 19 ± 9.4 vs B = 23 ± 6.7) (Fig. 4I). However, mosquitoes offered blood had higher intersip intervals when compared to those offered sucrose (S = 22 ± 7.9 vs B = 48 ± 18) (Fig. 4C). Moreover, the mean ingested volume of 10% sucrose solution (1.34 ± 0.16 µL) or blood (1.23 ± 0.24 µL) were not significantly different (Fig. 4J). Figure 4K shows the destination of sucrose or blood to the crop or midgut, respectively, and the relative concomitant enlargement of those organs that support the finding of equal meal volume ingested for sucrose and blood (Fig. 4J).

**Figure 5:**
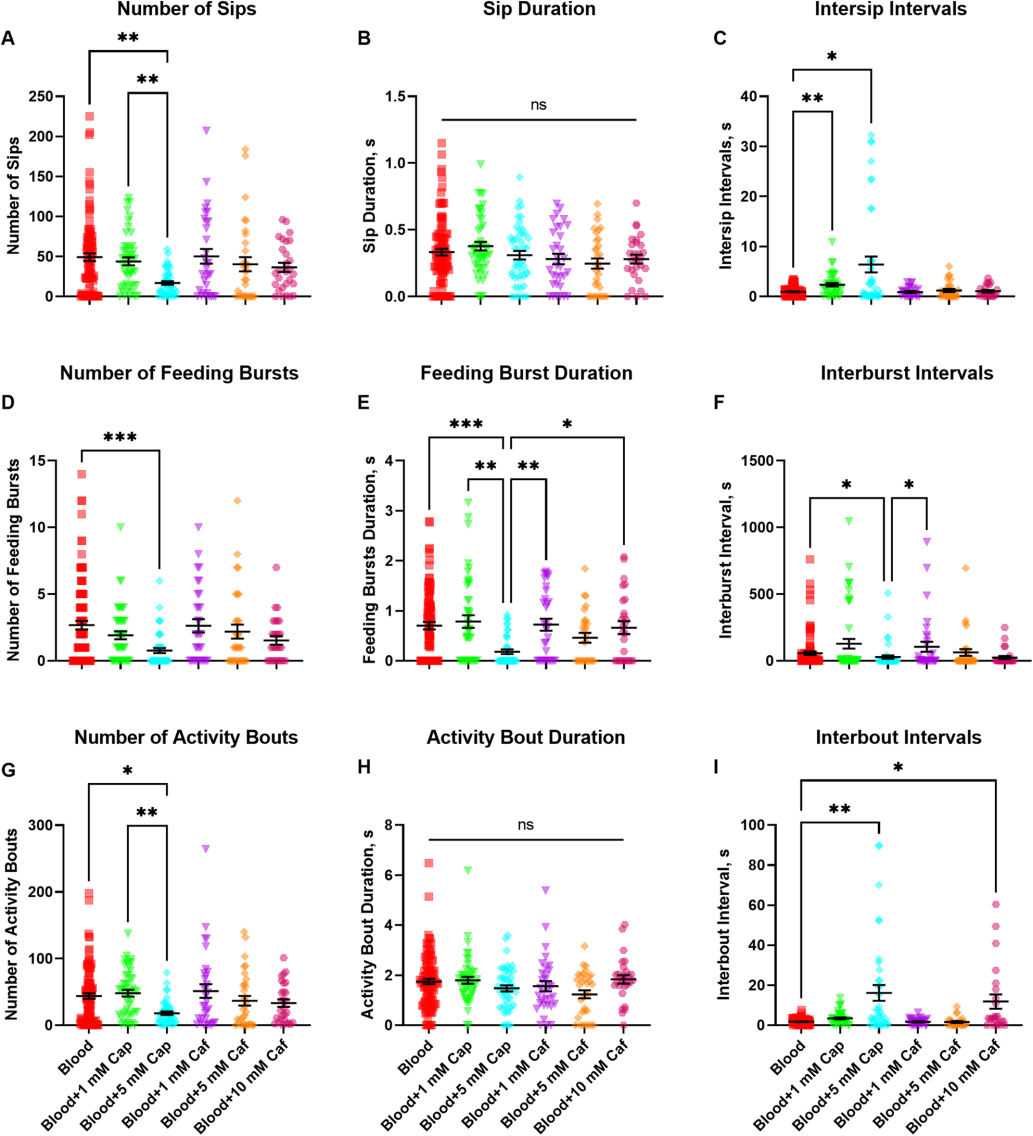
The effect of the addition of capsaicin or caffeine to the blood meal on feeding behavior outputs. Female *Aedes aegypti* (Liverpool strain) were offered blood (squares in red), blood containing 1 mmol L^*−*1^ capsaicin (inverted triangles in green, Blood + 1 mM Cap), blood containing 5 mmol L^*−*1^ capsaicin (diamonds in cyan, Blood + 5 mM Cap), blood containing 1 mmol L^*−*1^ caffeine (inverted triangles in purple, Blood + 1 mM Caf), blood containing 5 mmol L^*−*1^ caffeine (diamonds in orange, Blood + 5 mM Caf), or blood containing 10 mmol L^*−*1^ caffeine (hexagons in magenta, Blood + 10 mM Caf), in a non-choice assay using the flyPAD system. (**A**) Number of sips. (**B**) Duration of the sips in seconds; (**C**) Duration of intersip intervals in seconds. (**D**) Number of feeding bursts, characterized as three or more consecutive sips. (**E**) Duration of each feeding burst, in seconds. (**F**) Interburst intervals, in seconds. (**G**) Number of activity bouts, which represent how often the mosquito approaches the food. (**H**) Duration of the activity bouts, in seconds. (**I**) Duration of the interbout intervals, in seconds. Symbols represent outputs from individual females, horizontal lines are means (n: at least 30 females per treatment) ± standard error of the mean (SEM). Kruskal-Wallis multiple comparison test; asterisks denote a statistical significance, where one asterisk (*) indicates *P* < 0.05, two asterisks (**) indicates *P* < 0.01, three asterisks (***) indicates *P* < 0.001, and not significant (ns) indicates *P* > 0.05.

**Figure 6:**
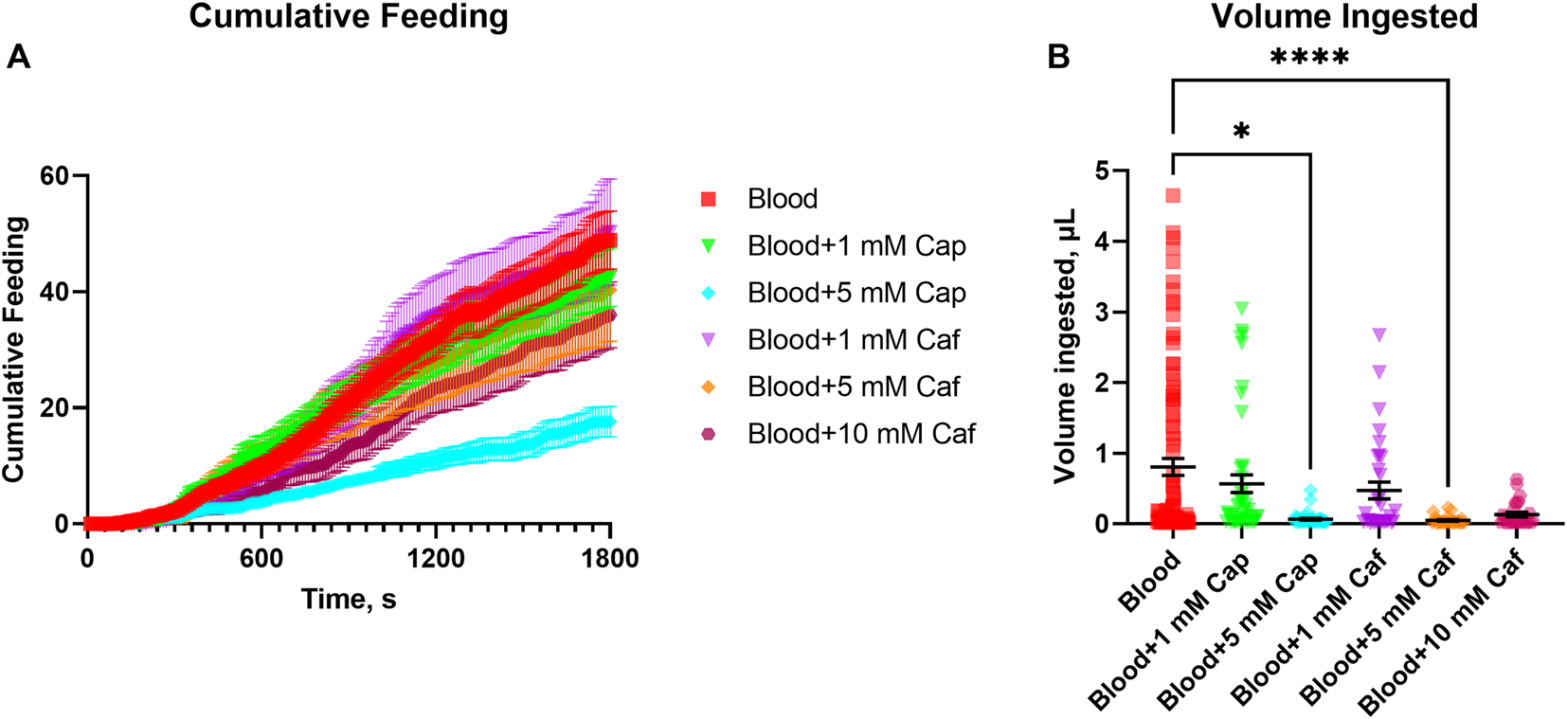
Effect of the addition of capsaicin or caffeine to the blood meal. Female *Aedes aegypti* (Liverpool strain) were offered blood (squares in red), blood containing 1 mmol L^−1^ capsaicin (inverted triangles in green, Blood + 1 mM Cap), blood containing 5 mmol L^−1^ capsaicin (diamonds in cyan, Blood + 5 mM Cap), blood containing 1 mmol L^−1^ caffeine (inverted triangles in purple, Blood + 1 mM Caf), blood containing 5 mmol L^−1^ caffeine (diamonds in orange, Blood + 5 mM Caf), or blood containing 10 mmol L^−1^ caffeine (hexagons in magenta, Blood + 10 mM Caf), in a non-choice assay using the flyPAD system. (**A**) Cumulative number of sips accounted by the flyPAD system for female mosquitoes during 30 min. The graph shows the cumulative average number of sips (number of contacts of the female proboscis with the sensor) per female when recorded every 10 s during 30 min. Symbols represent means (n = at least 30 replicates) ± standard error of the mean (SEM). Friedman’s tests followed by Dunn’s multiple comparisons test, *P *< 0.0001 for Blood vs. Blood + 5 mM Cap. (**B**) Volume of blood ingested, in microliters, calculated from the fluorescence contained in meals. Symbols represent means (n: at least 30 females per treatment) ± standard error of the mean (SEM). Kruskal-Wallis test, asterisks denote a statistical significance, where one asterisk (*) indicates *P* < 0.05, four asterisks (****) indicates *P* < 0.0001.

The flyPAD system was then validated for identifying compounds that potentially affect feeding behavior and/or meal size, such as feeding deterrents or repellents. For this, we performed a second non-choice assay in which females were offered in both arena channels blood containing ATP and fluorescein as control, or the same mix with either capsaicin at 1 mmol L^−1^ or at 5 mmol L^−1^, or caffeine at 1 mmol L^−1^, at 5 mmol L^−1^, or 10 mmol L^−1^ (Figs. 5 and 6). Capsaicin at 1 mmol L^−1^ did not decrease the number of sips with respect to the control blood meal. However, at 5 mmol L^−1^ it significatively reduced the number of sips performed by the females (17 ± 2.3) with respect to both capsaicin at 1 mmol L^−1^ (44 ± 5) and control blood meal (49 ± 4.8) (Fig. 5A), as well as reduced both the number of feeding bursts (0.77 ± 0.18) (Fig. 5D) and of feeding activity bouts (18 ± 2.5) (Fig. 5G), when compared to the control group (2.7 ± 0.32 and 44 ± 4.2, respectively). Similarly as for the number of sips, capsaicin at 5 mmol L^−1^ reduced the number of activity bouts with respect to capsaicin at 1 mmol L^−1^ (48 ± 5). Capsaicin at 5 mmol L^−1^ also reduced the feeding bursts duration (0.18 ± 0.05) with respect to both blood (0.7 ± 0.07) or capsaicin at 1 mmol L^−1^ (0.79 ± 0.13) (Fig. 5E). However, for interburst intervals duration only, capsaicin at 5 mmol L^−1^ (Fig. 5F) (28 ± 13) caused a significant reduction compared to the control group (58 ± 14). Capsaicin increased the intersip intervals both at 1 mmol L^−1^ (2.4 ± 0.4) and 5 mmol L^−1^ (6.4 ± 1.6) (Fig. 5C) but only at 5 mmol L^−1^ increased the interbout intervals (16 ± 3.9) with respect to blood (1.7 ± 0.2) (Fig. 5I).

Caffeine, on the other hand, at concentrations ranging from 1 to 10 mmol L^−1^ in the blood meal did not affect feeding behavior patterns with respect to the control blood (Figs. 5A–H), except for the interbout interval duration, which was significantly increased by caffeine at 10 mmol L^−1^ (12 ± 3.6) compared to blood (1.7 ± 0.2) (Fig. 5I). Neither capsaicin nor caffeine affected the sip duration (Fig. 5B) or the activity bout duration (Fig. 5H).

Observing the cumulative feeding behavior during 30 min, which consists of the cumulative average number of sips per female when recorded every 10 s during the duration of the assay, capsaicin at 5 mmol L^−1^ induced a significant change in the behavioral feeding kinetics (Fig. 6A) (*P* < 0.0001), with a reduced average number of sips per female. This resulted in a significantly reduced volume ingested with respect to the control blood (Fig. 6B), coupled with a reduced number of activity bouts and longer intersip intervals by these females (Figs. 5D,G), as expected and consistent with the correlations of variables and volumes obtained for blood (Table 1).

Despite the unchanged feeding behavior in the groups offered blood supplemented with caffeine regarding how often (similar number of sips, intersip intervals, number of feeding bursts and activity bouts) or for how long (feeding burst duration, interburst interval duration) they probed the arena channels, females offered caffeine at 5 mmol L^−1^ ingested a significantly smaller volume of the meal, indicating that this compound is likely a feeding deterrent but not repellent.

## Discussion

We successfully adapted the use of the flyPAD for the characterization of the feeding behaviors of female *Ae. aegypti* mosquitoes for sucrose solution and blood. Feeding variables referred to sips, feeding bursts and feeding bouts registering for each their number, duration, and interval. The flyPAD outputs reflected differential behaviors as the females discriminated meal quality in choice and non-choice assays. Further, the addition of a fluorescent tracer to the offered meals allowed to associate behaviors to the volumes ingested for water, sucrose solution, and blood with or without standard dipteran feeding deterrents.

We have not found any other report on the use of this system with dipterans other than *Drosophila*. Even though the system was originally designed to hold meals in agarose, it was adapted to hold liquid meals by sealing the bottom of the wells with qPCR plates sealing adhesive pieces. However, it is crucial for the adhesive pieces to be thoroughly pressed onto the bottom surface of the wells; any liquid leakage short-circuits the system causing the signal to go flat.

Our results indicate that females deprived from any macronutrients for 24 h probed more frequently and preferred to feed on sugar over water in choice assays, as anticipated (Figs. 2A,D,G). It appears that once the choice of sucrose solution was made, females fed almost continuously because the interbout intervals lasted only a few seconds (Fig. 2I). When drinking water, *Ae. aegypti* ingests small amounts, which are directly taken into the midgut, while when drinking nectar, larger amounts of liquid are ingested and are directed to the crop^44^. Whereas water does not provide any nutrients for mosquito survival, water intake is still very a common behavior in both male and female mosquitoes. Water helps avoid dehydration and maintain their water balance, especially in diapausing mosquitoes^45,46^.

For the mosquitoes to feed, the addition of the slide warmer placed under the chambers as a continuous heat source served twofold: allowed standardization of feeding conditions to compare feeding on sucrose solution or blood, which were performed simultaneously, and second, proved to be essential for feeding on blood, since the heat emitted by the endothermic vertebrates is one of the multimodal information used by hematophagous insects to localize and recognize potential hosts^47^. Without the heat source also the recovery time from anesthesia increased. Moreover, temperature has been proven to increase mosquito activity and ingestion of water and sucrose solution; observation of females at 30° C resulted in significantly higher flight activity and liquid intake, compared to 20 °C^48^. Furthermore, to avoid biases, mosquitoes were deprived of sucrose and blood prior to experiments, but not from access to water. Dehydration leads to both higher activity and increased blood feeding^49^, which would alter observed feeding behaviors and ingested volume.

FlyPAD allows for the analyses of several different feeding behavior aspects, as previously mentioned, such as sips, feeding bursts, defined as three or more consecutive sips, and feeding bouts, characterized as a set of two or more feeding bursts. The bouts, in return, are characterized as a sequence of two or more consecutive bursts^43^. The system also analyzes the durations of each of those behavioral variables, as well as timing the interval between proboscis-sensor or leg-sensor interactions. While in contact with the host, mosquitoes often perform exploratory bouts, during which they will frequently contact the host skin by walking and lightly tapping the puncture site with the labella^50^. Interestingly, despite being capillary feeding insects with piercing-sucking mouthparts, the female of *Ae. aegypti* fed to apparent repletion through open pool feeding on the flyPAD channels without the need of puncturing a membrane-like apparatus. The same behavior has been observed before for fasted females feeding on a crucible containing whole sheep blood^50^.

Aside from olfactometers, which investigate the responses of mosquitoes to odors^51^, other methods for blood feeding behavior analysis rely on the presence of a live host, as is the case for the bitometer^39^. The use of human voluntaries as bait for feeding behavior evaluation restricts the number of experiments that can be performed, aside from prohibiting the use of infected mosquitoes or testing different compounds, for example. Alternatively, the use of non-human mammals can also limit experimentation due to the need for ethics committee approval and costly laboratory animal maintenance. On the other hand, the biteOscope relies on an artificial feeding apparatus attached to mosquito cages and imaging, along with computerized automated characterization of the feeding behaviors^42^. Superior to these methods measuring bites, the Vectorchip is a microfluidic platform that allows high throughput genomic analysis of the mosquito saliva and pathogen transmission in a bite-by-bite manner^52^. The flyPAD system not only enables automated quantitative analysis of distinct behaviors but also facilitates the study of individual behavior, in addition to its capability of conducting choice assays and measuring the volume ingested by the addition of a tracer, such as fluorescein.

Mosquito feeding behavior is considerably heterogenous not only in the total number of bites, but also in the duration of these encounters, and both can be affected by external factors, such as temperature^53^. Characterizations through eletropenetrogram of the stylet probing behavior demonstrated that the bites of *Ae. aegypti* consist of a sequence of patterns described as surface salivation, stylet penetration through the skin, penetration of deeper tissues and location of blood vessels, and active ingestion^41^. The same study, however, also showed that even in highly standardized conditions, *Ae. aegypti* displayed behavioral plasticity, which resulted in a variation of performance of behaviors. This variation is likely the reason why, although the flyPAD outputs are consistent with observed behavior and calculated meal volumes ingested by the mosquitoes, the highest correlation coefficients among outpus were approximately 0.5 for both sucrose and blood (Table 1). Both the number of sips and the number of activity bouts demonstrated to be positively correlated to the meal volume ingested by the mosquitoes for both sugar solution and blood (Fig. 3), which means that these outputs are likely the most proportional and reliable when working with *Ae. aegypti*. Similarly, these two variables showed significative positive correlation with the food intaken by *D. melanogaster* adults^43^. In contrast to our findings, the activity bout duration correlated with meal volume ingested in fruit flies^43^, but not in females of *Ae. aegypti* (Figs. 3H, 4H).

It is obvious from correlation analyses between output variables and meal volume ingested (Fig. 3) that for all variables, the number of interactions with the blood channels (red squares) which did not result in measurable ingested blood volumes was higher than for those offered 10% sucrose solution, as can be observed by the higher number of red over blue squares on the Y axis but near zero microliters. These recordings represent instances in which females touched/probed the blood channels with their proboscis or legs but did not ingest. It must be noted that in all panels of Fig. 3 the data represent instances in which behaviors were observed, but not all females contributed to all output variables (i.e. did not perform a specific behavior); this explains the different number of observations (xy pairs) recorded for the different output variables (Table 1).

As the number of feeding bursts, feeding burst duration or interbust interval did not correlate with the ingested volume of blood (Figs. 3D–F), we speculated these variables may not be reliable when investigating the effect of blood feeding stimulants or deterrents on meal volume acquisition, which was proven true when testing caffeine (Figs. 5D–F). However, the behaviors could be different because indeed there were significant differences in the first two, number of feeding bursts and their duration (Figs. 5D,E) without resulting in differences in total volume ingested (Fig. 6B).

When comparing sucrose and blood feeding (Fig. 4), in non-choice assays, starved females ingested similar volumes of either blood or sucrose solution (Fig. 4J), even though females offered sucrose interacted more often with the meal, as evidenced by the higher number of sips and of feeding bursts, along with the longer average duration of feeding bursts and longer average intersip intervals (Figs. 4A,D,E,G). It appears that females ingested similar volumes of sucrose and blood by increasing the number of sips, the number of feeding bursts and their duration when feeding on sucrose, compensating for perhaps smaller volumes of sucrose acquired during each interaction. The viscosity of the feeding solution affects the sipping capability of the mosquito, which may result in different feeding responses, leading to dissimilar feeding behavior among females^54^. In laboratory conditions, in the absence of a blood-host stimuli, sugar feeding is frequent, but when the mosquito is exposed concurrently to both, sugar feeding practically ceases^55^. These authors also observed that there are only subtle differences in the general distributions of sugar and host feeding rhythms for different mosquito species^55^. Sugar feeding can confer major competitive advantage under deprivation circumstances, especially in the case of *Ae. aegypti*, a vector species which highly depends on sugar for survival^34^.

The flyPAD system proved to be a powerful tool in the identification of molecules that potentially affect mosquito feeding behaviors. Both caffeine and capsaicin are known to cause aversion in dipterans due to changes in the taste qualities of the meal^56–58^. We found that the flyPAD system distinguished behaviors that were influenced by the identity of the tastant (capsaicin vs caffeine) as well as their concentration.

Sucrose supplemented with at least 10 mmol L^−1^ of caffeine results in meal rejection by female *Ae. aegypti*^56^, at lower concentrations, i.e. 1 mmol L^−1^, caffeine does not elicit electrophysiological response and does not affect the meal ingestion^59,60^, which is consistent with our behavioral results. Similarly bitter tasting compounds, quinine and lobeline, also deter blood feeding in *Ae. aegypti* at 1 mmol L^−1^ and 5 mmol L^−1^, respectively, when supplemented directly into the blood meal^27^. The same compounds, however, do not significantly reduce engorgement when applied directly onto the host skin at tenfold higher concentrations^27^. In this study, caffeine reduced the volume of ingested blood (Fig. 6B) but did not decrease meal probing even at the highest concentration (Figs. 5A,B,D,E,G,H). Therefore, it is possible that this compound acts as a blood-feeding deterrent without eliciting an aversive response in the mosquitoes. The composition of blood with phagostimulants such as ATP, NaCl, sodium bicarbonate, and glucose coupled with the fact that the ATP receptor is located in the tip of the stylet in *Ae. aegypti*^38^, explains that caffeine does not prevent probing even when at the high concentration of 10 mmol L^−1^ (Fig. 5). The only significant difference for caffeine at 10 mmol L^−1^ was a longer interbout interval (Fig. 5I). Evidence suggests that the receptors involved in detecting bitter tastants are likely located in either the inner face of the labrum or in the cibarium of mosquitoes^61–65^. The internal sensors for caffeine may be highly sensitive in *Ae. aegypti*, like those for quinine in *Anopheles* mosquitoes^63^, because the females did not ingest the blood with caffeine. Based on these results, for research on feeding deterrents, it appears necessary to pair the detailed assessment of the variables provided by the flyPAD in conjunction with meal size quantitation methods, such as the addition of the fluorophore used here, to fully assess the effects of the tested compounds.

When *D. melanogaster* were presented with a choice between capsaicin-containing sucrose and plain sucrose, they exhibited a dose-dependent preference for capsaicin from 3 to 12 µmol L^−1^^66^. While *Anopheles stephensi* mosquitoes accepted a 10% sugar solution with 50 µmol L^−1^ capsaicin for two days, after blood feeding on mice the treated females showed a reduction in the number of eggs laid. The reduction in fertility was caused by capsaicin inhibition of the TOR signaling pathway^67^. *Ae. aegypti* females avoided feeding on capsaicin-supplemented ATP/salt solutions at higher concentrations of 1 and 5 mmol L^−1^^58^. In an equivalent manner, our results showed that these mosquitoes also avoided feeding on blood supplemented with capsaicin at 5 mmol L^−1^ (Fig. 5). Our found significant correlation of blood volume ingested with the number of activity bouts (Table 1) still held for blood containing capsaicin (Figs. 5G and 6B), which acts on external transient receptor potential (TRP) channels^68^ known to be expressed in the periphery^26,36,69,70^. The flyPad system showed that *Aedes* females were more sensitive at the periphery to capsaicin than caffeine (Fig. 5A). It is also obvious that capsaicin at 5 mmol L^−1^ significantly reduced the cumulative feeding with respect to the other treatments (Fig. 6, cyan trace). Whereas Fig. 5A shows the endpoint of the number of sips taken by each individual female, Fig. 6A depicts the average number of sips taken by all females at any point during the experiment, constituting the cumulative feeding. The latter allows for the observation of changes in the behavior pattern during feeding activity, i.e., a delayed start or a plateau.

Interestingly, even though capsaicin at 5 mmol L^−1^ induced changes in the feeding behaviors, i.e., reduced number of sips, bursts and bouts, there were no differences in the duration of the sips nor of the bouts (Fig. 5B,H). This is significant for our validation of the flyPAD for evaluating feeding deterrents when in blood, as these variables were not correlated with ingested volume either when testing sugar solution or blood only (Table 1).Comparably to our findings with capsaicin, although arboviral infection can significantly increase the number of unsuccessful bites conducted by the mosquitoes, it does not affect the average duration per probe performed^71^. This evidence and our results with the flyPAD system indicate that the least informative behavioral outputs for analyzing female mosquito feeding behavior are sip duration and activity bout duration (Table 1). They were not significantly different when comparing sucrose vs water (Fig. 2), sucrose vs blood (Fig. 3) or when comparing control blood to feeding deterrents at concentrations that affected at least some of the variables (Fig. 5).

For treatments that decreased the volume ingested such as water only (Fig. 2I) and concentrations of feeding deterrents (Fig. 6B), the increase in duration of interbout intervals appears informative (Fig. 5I). However, this variable was not significantly different when testing normal diets of sucrose solution and control blood and was also not correlated to ingested volumes of these two diets (Fig. 4I). Therefore, under the conditions of our experiments with *Ae. aegypti* females, “sip duration” and “activity bout duration” as detected by the flyPAD may not be influenced by taste peripheral input and may be either “hard-wired” at the central level and/or constrained by physiological control of the muscles involved or of other internal organs during feeding. Mosquitoes have a two-pump system located in their head, formed by the cibarial dilator pump and the pharyngeal dilator pump, which synergistically create a large pressure gradient allowing for rapid intake of sizeable amounts of blood^72^. The flow rate of liquid during the sip is constrained by the high resistance imposed by the small diameter of the proboscis making the pumping a very energetically demanding process^73^. Therefore, controlling the volume flow rate is a key factor for feeding performance in mosquitoes^74^.

Blood feeding is crucial for egg development in hematophagous species. Understanding mosquito blood feeding behavior is also crucial for understanding the pathogen transmission dynamics. Dengue virus infection not only increases mosquito attraction to hosts but decreases the biting efficiency, which, in turn, results in more bites to reach the same levels of engorgement observed in uninfected females^71^. Infection also leads to longer time spent probing and feeding, both contributing to the efficiency of *Ae. aegypti* as a vector of dengue virus^75^. The flyPad system as adapted herein could also be useful to quantify and more deeply analyze changes in feeding behaviors resulting from mosquito infection with this or other pathogens.

Provided with these adaptations, the flyPAD proved to be a reliable tool that can be used to detect alterations in both sugar and blood mosquito feeding behaviors induced by different treatments, as well as detect the effects of feeding deterrents, repellents, attractants, or stimulants added directly to the meals. At minimum it can contribute to the screening of what tastants can be detected by mosquitoes and register mosquito diet choices. FlyPAD is a semiautomated method that generates a large amount of standardized and unbiased behavioral data, which gives way to high-throughput biological activity testing. As shown here for the capsaicin and caffeine behavioral responses the flyPAD may help uncover or confirm differences in the mode of action of novel molecules and contribute to elucidate and correlate taste perception with behavior.

## Materials and methods

### Insect rearing

Mosquitoes of the Liverpool strain of *Aedes aegypti* (L.) were reared in cages (30.5 cm × 30.5 cm × 30.5 cm) in an incubator at 28 °C and approximately 80% humidity, with a 16/8 h light/dark light cycle. Ground fish food (Tetra, Blacksburg, VA) was provided throughout the aquatic larval stage. Larvae were kept in 1 L trays at low densities to ensure adults of homogeneous large size^76^. For adult stages, 10% (w/v) sucrose solution was supplied ad libitum in a covered cup with partially soaked dental braided cotton rolls.

Females 7 to 14-days-old were used for all feeding behavior bioassays. After adult emergence, females were only fed sucrose solution until 24 h prior to the experiments, when the cups containing sucrose solution inside the cages were replaced by cups containing water only.

### Solutions and meals preparation

For the fluorescent tracer added to the meals, either sugar solution or blood, a 0.2% (w/v) stock solution of aqueous fluorescein sodium salt (VWR Radnor, PA, USA, 0681-100G) was prepared^77,78^, and the tube was then wrapped in aluminum foil to avoid light exposure. All sugar meals were prepared as 10% sucrose and 0.002% (w/v) fluorescein as final concentrations. All blood meals were prepared using defibrinated sheep blood (HemoStat Laboratories, Dixon, CA, USA) with the addition of 1 mmol L^−1^ ATP and 0.002% (w/v) fluorescein. Adenosine 5′-triphosphate (ATP) disodium salt hydrate (Millipore Sigma, A2383) was reconstituted in 25 mmol L^−1^ NaHCO_3_ to a final concentration of 200 mmol L^−1^. The buffered solution minimizes ATP hydrolysis. This solution was then aliquoted and kept at − 20 °C.

To test the flyPAD sensitivity for detection of the influence of chemicals on the feeding behavior, two feeding deterrents^79^, caffeine (C0750, Sigma-Aldrich, St Louis, MO, USA) or capsaicin (M2028, Sigma-Aldrich) stock solutions at 100 mmol L^−1^ in ethanol were added to the blood meal to a final concentration of 1 or 5 mmol L^−1^. For caffeine evaluated at 10 mM, the reagent was reconstituted directly in the blood meal preparation to avoid excessive solvent. As a control, the highest volume of solvent was added to the control blood group, to a final concentration of 5% (v/v) of the total blood meal volume.

### FlyPAD bioassays

For the behavioral experiments, the flyPAD automated monitoring system of feeding behavior developed for adult *Drosophila* flies^43^ was adapted for female mosquitoes (Fig. 1). Briefly, each chamber has four independent arenas, and each arena contains two wells to be filled with different diets, as it was originally designed for choice assays (Fig. 1A). Each of these wells/diets is considered one channel. To adapt the system for mosquitoes the arenas were custom-made taller (approximately 8 mm instead of 6 mm Fig. 1B) by adding an extra horizonal plexiglass plate allowing standing female mosquitoes to walk and freely feed from either of the two wells. For the assays, the bottom of each flyPAD channel was sealed using a 1 cm^2^ piece of adhesive film for qPCR plates (VWR, 60941-078) (Fig. 1C). The behavioral chambers were then placed onto a slide warmer (Barnstead/Lab-Line, USA) and heated at approximately 37 °C for all feeding assays with water, sucrose solution or blood. Then, 4 µL of the meal were carefully pipetted into each channel (Fig. 1D). For choice assays, meals consisted of either water, or 10% sucrose offered in one of the channels of each arena. For non-choice assays, within each arena the same meal was offered in the two channels. Meals consisted of either 10% sucrose containing fluorescein or blood containing fluorescein and ATP at 1 mmol L^−1^.

After delivering the meal treatments to the channels, the arena’s lids were immediately closed to avoid excessive evaporation. For both choice or non-choice assays, females were anesthetized using humid CO_2_^80^ for 30 s and one female was transferred to each arena. Feeding behavior was recorded for 30 min starting immediately after one female was placed in each of the arenas.

To acquire the streamed capacitance data and simultaneously acquire data from video capture of the mosquito behavior from a selected sample arena, we used the Bonsai data stream processing package^43,81^. The software is available at http://www.flypad.pt. Video monitoring was performed using a Blackfly camera (FLIR Integrated Imaging Solutions, Inc., BFS-U3-16S2C-CS). All subsequent signal processing and data analysis steps were done in MATLAB (Mathworks Inc., Portola Valley, CA, USA).

A female “sip” is defined as the contact of the proboscis with the food, with the “sip duration” measuring the period from contact until the detachment of the proboscis from the food. A feeding burst is defined as three or more consecutive sips. A “feeding bout” is defined as a set of two or more consecutive feeding bursts^43^.

### Meal consumption quantification

The total volume of sucrose solution or blood ingested by each female was estimated using the fluorescein quantification method as described^77,78^. Briefly, after the 30 min assays, females were anesthetized using humid CO_2_ and individually transferred to 2 mL polypropylene tubes with screw caps and silicon O-rings, containing five 1.4 mm ceramic beads (Omni International, 19-645-3) and 100 µL of phosphate-buffered saline (PBS) (655090, Greiner Bio-One, Kremsmünster, Austria). Samples were then stored at – 20 °C in the dark until reading their fluorescence intensity, a step that can be postponed for one to two days after sample storage. For tissue disruption, samples were thawed and placed on a bead mill homogenizer (Omni BeadRuptor 12, Omni International) at speed 5.65 for 30 s. For every assay, either with sucrose solutions or blood treatments, respectively, a standard curve was prepared by adding 10 µL of the assay 10% sucrose solution, or blood containing 0.002% fluorescein and 1 mmol L^−1^ ATP to 390 µL of PBS, and in both cases, followed by a serial dilution at a 1:2 dilution rate. For each dilution, 100 µL were transferred to a 2 mL tube containing 1.4 mm ceramic beads and single females were homogenized as indicated above. Then, 20 µL of the homogenized samples were transferred to a 96-well black/clear bottom plate (Greiner, 655090) containing 180 µL of PBS. The fluorescence intensity was detected using a Clariostar plate reader (BMG Labtech, Ortenberg, Germany) at wavelengths 485/520 nm excitation/emission. The volumes of sugar or blood ingested by females were calculated as a ratio between the relative fluorescence units (RFU) corresponding to each female homogenate sample and the standard curve slope (RFU µL^−1^).

### Statistical analyses

Feeding behavior bioassay data were analyzed and graphs were produced using GraphPad Prism v9.5 software (GraphPad Software Inc., San Diego, CA, USA). Results were assessed for normality, but this was rejected, therefore, they were analyzed by Mann-Whitney test or Kruskal-Wallis one-way ANOVA followed by a corrected Dunn’s multiple comparisons test, and the results were presented as the mean ± standard error of the mean (SEM). Cumulative behavioral data were analyzed by repeated-measures one-way ANOVA followed by a nonparametric Friedman’s tests followed by Dunn’s multiple comparisons test; results were presented as the mean ± SEM. The strength of association between two variables was assessed through the nonparametric Spearman’s rank correlation test.

## Data Availability

The datasets used and/or analyzed during the current study available from the corresponding author on reasonable request.
